# Microbiota and Particulate Matter Assessment in Portuguese Optical Shops Providing Contact Lens Services

**DOI:** 10.3390/healthcare5020024

**Published:** 2017-05-15

**Authors:** Carla Viegas, Tiago Faria, Cátia Pacífico, Mateus Dos Santos, Ana Monteiro, Carla Lança, Elisabete Carolino, Susana Viegas, Sandra Cabo Verde

**Affiliations:** 1 GIAS, Escola Superior de Tecnologia da Saúde de Lisboa—ESTeSL, Instituto Politécnico de Lisboa, 1990-096 Lisbon, Portugal; tiago.faria@estesl.ipl.pt (T.F.); catia.s.pacifico@gmail.com (C.P.); mateus.santos2094@gmail.com (M.D.S.); ana.monteiro@estesl.ipl.pt (A.M.); carla.costa@estesl.ipl.pt (C.L.); et.carolino@estesl.ipl.pt (E.C.); susana.viegas@estesl.ipl.pt (S.V.); 2 Centro de Investigação em Saúde Pública Escola Nacional de Saúde Pública, Universidade Nova de Lisboa, 1600-560 Lisbon, Portugal; 3 Centro de Ciências e Tecnologias Nucleares, Instituto Superior Técnico, Universidade de Lisboa, 2695-066 Loures, Portugal; sandracv@ctn.tecnico.ulisboa.pt

**Keywords:** indoor air quality, microbiota, particulate matter, assessment, optical shops, contact lenses, bioaerosols

## Abstract

The aim of this work was to assess the microbiota (fungi and bacteria) and particulate matter in optical shops, contributing to a specific protocol to ensure a proper assessment. Air samples were collected through an impaction method. Surface and equipment swab samples were also collected side-by-side. Measurements of particulate matter were performed using portable direct-reading equipment. A walkthrough survey and checklist was also applied in each shop. Regarding air sampling, eight of the 13 shops analysed were above the legal requirement and 10 from the 26 surfaces samples were overloaded. In three out of the 13 shops fungal contamination in the analysed equipment was not detected. The bacteria air load was above the threshold in one of the 13 analysed shops. However, bacterial counts were detected in all sampled equipment. Fungi and bacteria air load suggested to be influencing all of the other surface and equipment samples. These results reinforce the need to improve air quality, not only to comply with the legal requirements, but also to ensure proper hygienic conditions. Public health intervention is needed to assure the quality and safety of the rooms and equipment in optical shops that perform health interventions in patients.

## 1. Introduction

Contact lenses are used for refractive correction, cosmetic enhancement, and other therapeutic reasons [1]. The number of contact lens wearers worldwide have been estimated as high as 140 million in 2005 [2]. Although no definite statistics are available regarding contact lens use in Portugal, an increasing number of the Portuguese population using soft contact lenses is observed. An international survey of contact lens prescribing for presbyopia shows that Portugal is one of the countries with higher levels of prescriptions (79%) for this condition alone [3]. The prevalence of lens-related complications is rising and is reported to be between 20.58% [4] and 50% [5]. Young males [2] are reported as the most affected by lens-related complications. The presence of bacteria, protozoa, and fungi on contact lenses predispose the patient to infections [6]. Different pathogenic organisms have been identified following the introduction of soft lenses in 1970s, with staphylococci and pseudomonas being the most common [4,6,7]. Microbial keratitis has a low prevalence, but is a serious condition that may be associated with hospital admission, time off from work, increasing the cost of medications and back-up spectacles [4]. This condition in contact lens wearers is mainly a bacterial process. However, *Acanthamoeba* have been associated with contact lens-related infections [4]. Fungal infections are uncommon, though some cases of *Fusarium* keratitis have been reported [8,9,10]. In addition, particle matter may serve as a favourable medium for the persistence of numerous species of fungi and bacteria, which may release allergens and toxins that exert different health effects [11], and may also be a vehicle for microbiota resuspension and dispersion [12].

There are no universal guidelines regarding the decontamination of ophthalmic instruments [13]. Infections may be transmitted from patient to staff or staff to patients by direct contact (with tears and mucous membranes), aerosol formation, or contamination of equipment [14].

Airborne microorganisms (a part of bioaerosol composition) can originate not only from humans (including patients), but can also be disseminated by diverse indoor characteristics (ventilation, equipment, and materials) and outdoor environmental sources [14,15,16,17,18]. In addition, surfaces (walls and floors) can be a deposit of nutrients and, consequently, potentiate microorganism proliferation that can be dispersed in air due to different activities [19].

In Portugal, orthoptists and optometrists manage the majority of contact lens adaptations at optometric clinics or optical shops within the private sector. In 2013 the number of optical shops in Portugal was 1540 [20].

Recent Portuguese legislation established new limit values for microbiological air load in indoor environments [21], replacing the previous legislation [22]. In the previous legislation, a critical limit of 500 CFU/m^3^ was defined as the threshold for fungi and bacteria concentration. Currently, the legal compliance also defines different evaluations regarding microorganism identification. For fungi, indoor concentrations should be less than outdoor concentrations; and for bacteria, the indoor concentration should not exceed the outdoor concentration by 350 CFU/m^3^. However, the critical limit of 500 CFU/m^3^ was applied in guidelines and other studies [23,24,25].

The same regulation presents two references for particulate matter, namely for PM10 (particles with a nominal mean aerodynamic diameter ≤10 μm) and PM2.5 (particles with a nominal mean aerodynamic diameter ≤2.5 μm) in indoor environments. PM10 delineates a subset of inhalable particles (referred to as thoracic particles) that are small enough to penetrate the respiratory tract (e.g., tracheobronchial region) and PM2.5 as an indicator for fine particulate matter based on consideration of particle penetration into the gas-exchange region [26]. Current regulation has an important difference from the previous version [22] using PM2.5 as a reference, because epidemiologic health studies have reported various health effects associated with particles with lower diameter. Therefore, PM2.5 has a smaller and stricter reference value (25 µg/m^3^) than PM10 (50 µg/m^3^).

The aim of this study was to assess microbiota (fungi and bacteria) and particle matter in optical shops. Microbiota assessment intended to cover indoor air, ophthalmic instruments, and surface contamination in thirteen optical shops in order to estimate the potential microbiological hazards for the patients and users of these services and health professionals. We also intend to determine the guidelines and legal compliance of the optical shops assessed, contributing to a specific protocol to ensure a proper microbiota and particulate matter assessment.

## 2. Materials and Methods

### 2.1. Optical Shops Assessment

A descriptive study was conducted between October 2015 and March 2016, in a total of 13 optical shops from the Lisbon area in Portugal. Ethical standards for the study complied with the Lisbon School of Health Technology requirements (optical shop consent and a declaration of anonymity and confidentiality). A walkthrough survey and checklist was applied in each shop in order to understand the hygiene and disinfection measures taken, the amount of workers present in the shops, the number of clients assisted prior to the microbiota sampling, the presence/absence of a heating ventilation and air conditioning (HVAC) system and other cleaning habits. It is important to note that the majority of the shops assessed did not have the HVAC systems working and in the street shops, the ventilation was provided only through the open door. All of the sampling and measurements were done at the same time of a normal working day.

### 2.2. Sampling

Air samples consisted mainly of four indoor samples (two for fungi and two for bacteria) and one outdoor sample in each optical shop, to be used as a reference. Air samples of 250 L were collected through an impaction method with a flow rate of 140 L/min (Millipore air Tester, Millipore, Billerica, MA, USA) onto each plate according to manufacturer’s instructions. Two different culture media were used in order to enhance the selectivity for bacterial and fungal populations growth: malt extract agar (MEA) supplemented with chloramphenicol (0.05%) was used for fungi and tryptic soy agar (TSA) supplemented with nystatin (0.2%) was applied for to assess the bacterial load. The air sampling plan followed the guidelines of the national legislation [21].

Surface samples were collected by swabbing corresponding indoor sites with a 10 cm × 10 cm square stencil, disinfected with a 70% alcohol solution between samplings, in line with the requirements [27]. Equipment swab samples were also collected side-by-side. The samples (Table 1) were sealed with parafilm and transported to the laboratory in a cooler bag. All of the collected samples were incubated at 27 °C for 5–7 days (fungi) or at 30 °C for seven days (bacteria). After laboratory processing and incubation of the samples, quantitative (colony-forming units—CFU/m^−3^ and CFU/m^−2^) results for fungi and bacteria were obtained, with the exception of samples collected from the assessed equipment. In this last case, prevalence was achieved through the isolate number of each species identified.

### 2.3. Fungal Identification

Fungal identification was achieved through macro- and microscopic characteristics, as described by Hoog et al. [28]. Macroscopic identification relied in the colony characteristics (e.g., colour, shape, and elevation) and was coupled with microscopic identification by performing microscopic mounts using tease mount or Scotch tape mount and lactophenol cotton blue mount procedures for microscopic identification of the fungal genera (or species, when possible). The prevalence of each fungus was calculated based in the number of isolates obtained from each genera/species/complex and the total number of fungi identified by site or by type of sampling (air, surface, or equipment).

### 2.4. Particulate Matter and Temperature and Humidity Assessment

Measurements of particulate matter (PM) were performed using portable direct-reading equipment (Lighthouse, model 3016 IAQ, Fremont, CA, USA) that gives information regarding mass concentration (mg × m^−3^) in five different sizes (PM0.5, PM1, PM2.5, PM5, PM10). Additionally, data related with particle number concentration by each diameter size were also obtained with the same equipment. In this case, particles results were given in six different diameter sizes, namely: 0.3 μm, 0.5 μm, 1 μm, 2.5 μm, 5 μm, and 10 μm. As mentioned in some literature, these data were also collected because they might be more closely correlated with adverse PM health effects [29,30]. One measurement with the duration of 5 min was done in each sampling as indicated by Portuguese legislation, and the results were obtained by calculating the average for each sampling period. This period of time was considered representative of the type of occupancy and tasks developed.

Simultaneously to the particulate matter assessment, temperature and relative humidity were also monitored through the same equipment and according to the International Standard ISO 7726:1998.

### 2.5. Data Analysis

Statistical software SPSS V22 was applied for statistical analysis. The data analysis was performed using univariate descriptive statistics with frequency (*n*; %), median, and graphical representations appropriate for the nature of the data. The results were considered significant at a 5% significance level. The Shapiro-Wilk test was applied to test data normality. To study the relationship between fungal load, bacteria load, and particle matter, and also to study the previous environmental variables’ relationship with temperature and relative humidity, the Spearman correlation coefficient was applied, since the normality assumption was not verified (*p* < 0.05). To compare fungal, bacteria load, and particle size concentration between the optical shops that use brooms for cleaning purposes and the ones that do not, a Mann-Whitney test was applied.

## 3. Results

### 3.1. Walkthrough Survey and Checklist

The collected data allowed obtaining crucial information to identify potential contamination sources from the indoor environment. Among the 13 optical shops, two (15.4%) were located inside a shopping mall; ten (76.9%) have daily cleaning intervention, and seven (53.8%) applied a broom as a cleaning measure. Moreover, it was found that an external company ensured indoor cleaning in four (30.8%) of the optical shops, and all of the health professionals confirmed that the optical equipment was disinfected with alcohol (with the exception made to pupilometers) between patients and hand-washing was ensured between patients in 11 (75.6%) optical shops. Only three shops (23.1%) have the HVAC system turned on and five (38.5%) did not have a washstand specific for the optometry office and all had manual opening.

There are no universal guidelines that apply to the decontamination of ophthalmic instruments. National disinfection protocols recommend the use of ethyl alcohol at 70% for surface disinfection, although this is a general recommendation for all health services. Additionally, there is no mandatory training for the health professionals working at optical shops, as this type of organization is not regulated as part of the health system in Portugal.

### 3.2. Fungal and Bacteria Load

**Figure 1:**
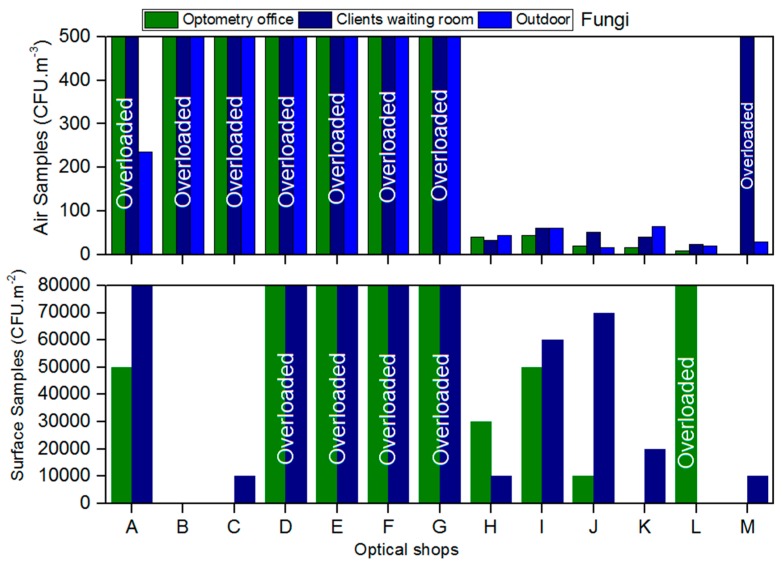
Fungal load present in the air and on surfaces from the assessed optical shops.

Air fungal load in client/patient rooms ranged from 24 CFU/m^3^ to >500 CFU/m^3^ and in optometry offices from <1 CFU/m^3^ to >500 CFU/m^3^ (Figure 1). Eight of the 13 shops analysed were above the threshold recommended in the guidelines for fungal air load (>500 CFU/m^3^). In ten shops, the indoor fungal load was higher than the outdoors, surpassing the legal requirement (Figure 1).

On the surfaces it was estimated that the fungal load ranged from <1 CFU/m^2^ to 7 × 10^6^ CFU/m^2^ in client/patient rooms and from <1 CFU/m^2^ to 5 × 10^6^ CFU/m^2^ in optometry offices (not considering the overloaded samples). Ten from the 26 surfaces samples were overloaded (>500 CFU/m^2^) and impossible to count colonies (Figure 1).

Evidence of fungal contamination was also found in equipment samples. From the total of the equipment sampled, only three of the 13 shops showed no sign of fungal contamination.

**Figure 2:**
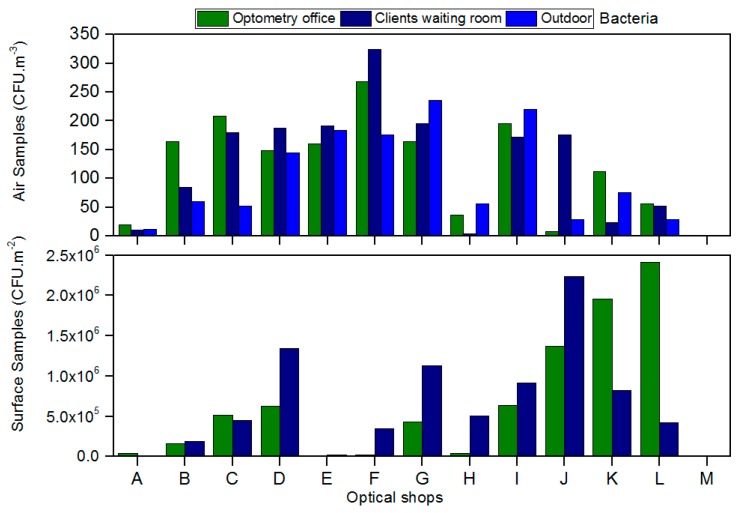
Bacteria load present in air and on surfaces from the optical shops assessed.

Regarding the air bacteria load, concentrations ranging from 4 CFU/m^3^ to 324 CFU/m^3^ in clients/patients room and from 8 CFU/m^3^ to 276 CFU/m^3^ in optometry offices (Figure 2) were found. One of the thirteen shops analyzed was above the threshold detailed in the legal requirement for bacteria air load (indoor concentration should not exceed the outdoor concentration by 350 CFU/m^3^) (Figure 2). Concerning the surfaces, the bacterial counts indicated to be between 1 × 10^4^ CFU/m^2^ to 2.24 × 10^6^ CFU/m^2^ in clients/patients room and 1 × 10^4^ CFU/m^2^ to 2.41 × 10^6^ CFU/m^2^ in optometry offices from (Figure 2). The presence of bacteria was also detected in every piece of equipment sampled in all of the shops.

### 3.3. Fungal Identification

The fungal diversity present in the samples was characterized based on the morphological features of the colonies (Table 2). Indoor and outdoor air were very similar regarding the mycobiota present, since these samples were highly colonized by fungi belonging to the *Alternaria*, *Cladosporium*, and *Penicillium* genera. Overloaded plates with *Chrysonilia sitophila* isolates were also found in three of the 13 samples of indoor and outdoor air. Fourteen different fungi genera/sections/species were isolated in air, with *Alternaria* sp. being the most prevalent (54.3%), followed by *Penicillium* sp. (14.6%) and *Cladosporium* sp. (14.0%). In addition to the most prevalent and *C. sitophila*, other fungi were identified, such as, *Talaromyces* sp., *Aureobasidium* sp., *Mucor* sp., *Geotrichum* sp., *Rhizopus* sp., *Acremonium* sp. and *Phoma* sp. Among *Aspergillus* genus, sections *Circumdati* and *Fumigati* were found in low counts (Table 2).

The most prevalent fungi found in the surface samples were *Aspergillus* section *Versicolores* and *Penicillium* sp. (36.4%), followed by *Aspergillus* section *Fumigati*, *Scytalidium hyalinum*, and *Cladosporium* sp. (9.1%). The presence of overloaded plates with *C. sitophila* isolates was also verified on the surfaces, together with *Phoma* sp. and *Rhizopus* sp. (Table 2).

*Aspergillus* section *Versicolores* and *Scytalidium hyalinum* were only isolated on surfaces (Table 2).

On the ophthalmic equipment, the most prevalent fungi were *Penicillium* sp. (76.2%), *Acremonium* sp. (9.5%), and *Rhizopus* sp., *Alternaria* sp., and *Cladosporium* sp. (9.1% each). Five samples also revealed the presence of *Chrysonilia* sp. at a number of isolates impossible to count (Table 2).

### 3.4. Particulate Matter

**Figure 3:**
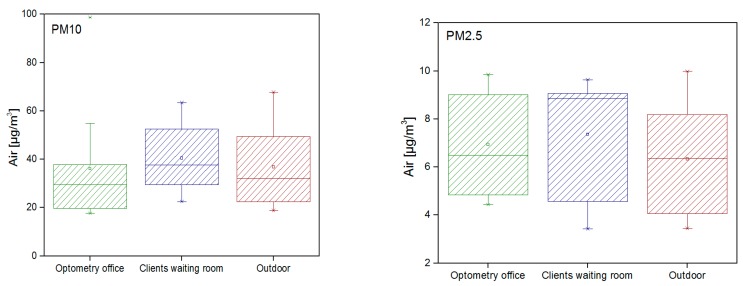
PM results in the different clinic areas.

As mentioned previously, results for two aerodynamic diameters were obtained (PM10 and PM2.5) (Figure 3). Results showed that clients waiting area had higher values for PM10 when compared with optometry office and outdoors (*p* > 0.05). For PM2.5, although with higher values, no statistically significant differences between samples were found.

### 3.5. Data Correlation Analysis

The correlation between microbiota assessment in air, and on surfaces and equipment is analysed in Table 3. Fungal load in air, and on surfaces and equipment presented moderate or strong positive correlation that could indicate that when fungal load in air increases, the same could occur on surfaces and equipment. A similar trend was observed in bacteria load assessment. However, a negative correlation (moderate to strong) between fungi and bacteria was found, suggesting that high fungal load is correlated with low bacteria load. According to the performed data analysis, fungi and bacteria were not correlated with the particulate matter assessment. However, a significant negative correlation was found between temperature and particulate matter concentration (PM2.5) (rs = −0.760; *p* = 0.011), air fungal load (Rs = −0.693; *p* = 0.026), and bacterial concentration on surfaces (rs = −0.778; *p* = 0.008). These results indicate that the higher the temperature, the lower the particulate matter concentration (PM2.5), air fungal load, and bacterial concentration on surfaces. Regarding the relative humidity no significant correlation was detected.

No statistically significant differences were detected in both fungal and bacteria contamination (air and surface) and particle size compared to the information obtained from the walkthrough survey and checklist.

Particulate matter assessment showed that an increase in a specific room could influence all of the other rooms of the optical shops with a similar increase.

The analysis between microbiota assessment and data obtained through the verification list, show a statistically significant difference, with optical shops that use a broom showing a higher fungal and bacteria load. No statistically significant differences were found between microbiota and particulate matter assessment and optical shops that presented a specific washstand and the ones that did not.

## 4. Discussion

Health professionals should ensure that their patients and staff are not exposed to infection risk while attending or working at their practice [13]. Optical shops must follow hygienic procedures to allow this specific environment presents adequate conditions for patient care. With a better understanding of the variables that influence the microbiota in optical shops effective control strategies can be established to reduce exposure risks of patients and, consequently, of vision health professionals [31].

It is suggested that indoor fungal levels should be compared with those found outdoors, since the first are dependent on the second [21,31,32]. Quantitative values of fungi were found to be equal (1/13) or higher (10/13) than outdoors in optical shops, suggesting fungal contamination sources from within and/or a concentration effect of fungi from outside to indoors. Additionally, in these shops there was no compliance with the national legal requirement and in seven of these 10, even with the previous legal requirements [21,22]. All of the overloaded plates were considered to have a higher load than 500 CFU/m^3^ due to health protection reasons.

Regarding the air bacteria load, most of optical shops indicated higher concentrations indoors than outdoors, suggesting bacterial sources from indoors, but only one store did not comply with the legal norms [21].

Air fungal identification was used to characterize the fungal burden present indoors, but also to verify the legal compliance. *Aspergillus* sections were isolated, namely *Fumigati* in two shops, and *Circumdati* in one shop, and in all situations surpassing the legal requirement of less than 12 CFU/m^3^. This value was set due to the toxigenic potential from both *Aspergillus* sections that are able to produce, among others, mycotoxins, gliotoxin, and ochratoxin A, already reported in different indoor environments [33]. These species may pose high clinical relevance and should not be underestimated, since they could constitute a major risk for health in humans and animals [34,35]. Moreover, according to the American Industrial Hygiene Association [36] in the *Field Guide for the Determination of Biological Contaminants in Environmental Samples*, the confirmed presence of the *Aspergillus* section *Fumigati* requires implementation of corrective measures.

Approximately 2–6% of the general population in developed countries is allergic to fungi and the higher sensitivity is detected with respect to genera of *Alternaria*, *Cladosporium*, *Aspergillus*, *Penicillium*, and *Fusarium* [37]. Two of these genera, *Cladosporium* and *Penicillium*, were found in all of the samples analysed in this study. Additionally, *Alternaria* (present in indoor and outdoor air samples) and *Aspergillus* (present in two optical shops) that were found in the present study are considered the most common allergens responsible allergic rhinitis and for 5–10% of asthma cases [37]. The results of the present study show that there is the need for public health intervention assuring the quality and safety of the rooms and equipment in optical shops that perform health interventions in patients.

Fungal load from surfaces present a higher range than in air samples, where the isolates were possible to count. In addition, *Aspergillus* section *Versicolores* and *Scytalidium hyalinum* were only isolated in surfaces and were not found in air. This result highlights the need to also collect samples from surfaces besides air in this setting, to ensure a more complete fungal contamination assessment [38].

In nine of the 13 trial frames assessed, the fungi species were identified, always being the equipment with higher fungal load in the analysed optical shops. Although all the health professionals confirmed that the disinfection of optical equipment was performed with alcohol (with the exception made to the pupilometer) between patients, the results do not comply with such disinfection. Trial frames regularly come into contact with patients during the refractive evaluation. The obtained results point out that disinfection is not done between every patient as reported or the disinfection procedures are not being done properly. This warrants further research to deepen the cleaning procedures and frequency of such cleaning.

The prevalence of fungal isolates are impossible to count with fast growing rates, such as *Chrysonilia sitophila*, *Phoma* sp., and *Rhizopus* sp., being among other drawbacks from classical-culture methods already reported [25], justifying the complementary use of molecular tools. Other studies already applied this strategy aiming to overcome conventional method limitations [38,39,40,41,42]. In addition to using conventional methods for fungi and bacteria applied to air samples that allows verification of legal compliance, analyses of surface samples applying molecular tools to target for specific indicators of harmful fungal and bacteria contamination in this specific environment should also be used. *Fusarium* sp. and *Paecilomyces* sp. [9,10,11,43] should be targeted due to reported clinical outcomes, being that the *Fusarium* genus is also a common contaminant of contact lens solution [44]. The *Aspergillus* genus should be also considered since it is broadly distributed in nature, with a large number of species frequently causing opportunistic infections and different clinical manifestations and diseases [45]. Among *Aspergillus* and considering not only their potential health effects, but also their environmental significance when detected indoors, the sections *Fumigati* [36,37,46,47,48], *Flavi* [36,37,46,48], *Terrei* [36,49], and *Versicolores* [35,36,48] should be adopted as indicators in optical shops.

The presence of the *Aspergillus* genus can also be harmful to the ocular system, as leukocyte defence is one of the ocular defence mechanisms that possess the ability to ingest and kill microorganisms. The absence of polymorphonuclear leukocytes is associated with fungemia with *Candida*, *Aspergillus*, and *Fusarium* spp. [50]. Eye trauma is the cause of fungal keratitis in temperate areas and the common fungal genera involved are *Fusarium*, *Alternaria*, and *Aspergillus*. The presence of *Aspergillus* in two optical shops is a public health concern, as *Aspergillus* species are the second most-common cause of fungal endophthalmitis [50].

In indoor air, humans and animals are assumed to be the main sources of bacterial aerosols, but these may also be created by disturbing previously-settled dust and in HVAC drainage systems [47]. Moreover, the building conditions, the level of occupation [51], and human activities are also responsible for bacterial concentrations [19,52,53]. *Staphylococcus* spp., besides, being reported as an infection agent following the introduction of soft lenses [4,6,7], is also abundant in indoor air [36,54]. Additionally, *Pseudomonas* is referred to as the most abundant and frequently-detected bacterium in the hospital context [55]. For future assessments, both genera could be regarded as potential bacterial indicators in optical shops.

Viable bioaerosol particles constitute a small percentage of the total concentration of microorganisms [56] and, therefore, a bias about microbiota in all optical shops assessed should be considered. To decrease the risk of infection several procedures of disinfection, sterilisation, and reprocessing should be made by eye care professionals. Cleaning of ophthalmic instruments is an essential prerequisite, as organic material (mucus, tears, skin, or make-up) may harbour infective organisms in dangerous concentrations and prevent adequate disinfection or sterilisation (insoluble deposits may require utilisation of isopropyl alcohol) [11].

Sampling the total air concentration of particulate matter only allows a simple estimate of exposure or indoor contamination that may not correlate with observed health effects [57]. The present study obtained data related with two aerodynamic diameters, allowing a more detailed risk assessment for patients and workers. Regarding particulate matter results, the client/patient waiting room showed higher contamination for both aerodynamic diameters. This fact is probably due to different aspects, namely a higher number of persons when compared with the optometry office promotes the transport and resuspension of particles to direct contact with the outdoors due to constantly open doors, and to an indoor lower dilution when compared with the outdoor environment [12]. This last aspect is also promoted by the lack of mechanical ventilation in the assessed optical shops. The importance of the mechanical ventilation to guarantee indoor air quality is well demonstrated in previous studies, particularly with particle contamination [58,59,60,61,62]. In the present study, the particulate matter assessment indicated that when these increase in a specific room all of the others rooms show the same tendency. This could be related to three different factors, specifically: there is no particle emission source in any specific location and, therefore, particles are coming, essentially, from the outdoors [12]. A previous study developed in Portugal already reported the influence of the outdoors in the contamination of indoor environments by nanoparticles [63]; particles are resuspended due to the movement of workers and patients [64,65,66]; and particles accumulate indoors because of the low ventilation rates, since there are no mechanical ventilation systems to guarantee dilution and dispersion [59,60,61,62,63]. Corroborating with our results, other authors reported no significant correlations between fungal and bacterial air load and PM concentrations, stating low concentrations of microorganisms associated with PM [67]. The attributed explanation was related to the influence of anthropogenic activities and atmospheric changes, and to the association of a large portion of bacteria with dust particles. Although fungi and bacteria correlated negatively, that could be due to competition reasons between microorganisms [68], and it was verified that fungi and bacteria air loads were influencing all of the other surface and equipment microbial concentrations. This situation supports the need to improve air quality, not only to comply with the legal requirements, but also to ensure proper hygienic conditions for this specific setting. Additionally, broom use, as in other health settings, should be avoided due to the increased contribution for fungal and bacteria contamination. This result reinforces that human activities, besides other factors, are important environmental variables that might influence microbial growth in hospitals [14] or other healthcare settings.

The negative correlation between temperature, air fungal load, and bacterial surface contamination was not consistent with what is expected, since a strict correlation between microbiota and temperature was already reported [69,70,71]. This may be justified by the effect of other environmental variables, such as workers and patients who may carry a great diversity of microorganisms [72], as well as the developed activities that may also affect fungal and bacterial load [71,73]. Regarding the negative correlation found between temperature and particulate matter, particularly PM2.5, it is important to mention that others aspects could influence the PM2.5 results, such as the number of occupants during the measurements and room size [74].

According to the data obtained, the high concentration of fungal and bacteria in air, on surfaces, and equipment sampled, must raise the awareness to the need of devising guidelines specific for this setting, since they can become an infection source. In addition, national legislation does not consider the occupants’ susceptibility, nor even the specificity of tasks that are developed in a specific environment, since the legislation is applied to several types of establishments, such as schools, offices, and hospitals, among others [40]. Considering this fact, the results should be compared with a more demanding threshold, such as the ones applied in hospital settings (200 CFU/m^3^) for fungi defined by Krzysztofik in 1992 [75]. Considering all of the data obtained, mechanical ventilation systems should be implemented in optical shops to avoid particulate matter entrance by open doors [58,59,60,61,62] and, consequently, deteriorate indoor air quality. Furthermore, microbiota data should be used to clearly define specific air quality guidelines for optical shops, and also procedures for surfaces and equipment cleaning. To the best of our knowledge, this is the first study developed in this specific setting and can be the first step towards a future protocol to ensure the proper microbiota and particle matter assessment in optical shops.

## 5. Conclusions

This work fills a gap providing information on the microbiota background and particulate matter in optical shops that provide contact lens services, and also their compliance with legal requirements. This study suggests the indicators that are representative of harmful fungal and bacterial contamination contributing to a future protocol to properly assess these pollutants. The confirmed presence of the *Aspergillus* section *Fumigati* requires implementation of corrective measures. Further investigations regarding sources of biological pollutants would be important to provide information to public health stakeholders.

## Figures and Tables

**Table 1:** Sampling sites (air and surfaces) and equipment assessed.

Air	Surfaces	Equipment
Clients/patients waiting room	Clients/patients waiting room floor	Trial frames
Optometry office	Optometry office floor	Foropter
Outdoor (reference)		Biomicroscope
		Pupilometer
		Automatic refractometer

**Table 2:** Fungal diversity present in the samples from the optical shops and respective prevalence (not considering the overloaded plates with the number of colonies impossible to count).

Samples	Fungi Identification	Prevalence (*n*; %)
Indoor air	*Alternaria* sp.	788; 54.3
	*Penicillium* sp.	212; 14.6
	*Cladosporium* sp.	204; 14.0
	*Aureobasidium* sp.	92; 6.3
	Others	156; 10.7
Outdoor air	*Alternaria* sp.	832; 56.5
	*Cladosporium* sp.	432; 29.3
	*Penicillium* sp.	108; 7.3
	*Exophiala* sp.	36; 2.4
	Others	64; 4.4
Surface	*Aspergillus* section *Versicolores*	40,000; 36.4
	*Penicillium* sp.	40,000; 36.4
	*Cladosporium* sp.	10,000; 9.1
	*Aspergillus* section *Fumigati*	10,000; 9.1
	*Scytalidium hyalinum*	10,000; 9.1
Ophthalmic equipment	*Penicillium* sp.	16; 76.2
	*Acremonium* sp.	2; 9.5
	*Rhizopus* sp.	1; 4.8
	*Alternaria* sp.	1; 4.8
	*Cladosporium* sp.	1; 4.8

**Table 3:** Correlation between microbiota assessment in air, and on surfaces and equipment.

	I	II	III	IV	V	VI	IX	XI	XV	XVI
I	-	-	-	-	-	-	-	-	-	-
II	0.946 **	-	-	-	-	-	-	-	-	-
III	−0.362	−0.383	-	-	-	-	-	-	-	-
IV	−0.323	−0.546	0.301	-	-	-	-	-	-	-
V	−0.421	−0.557 *	0.581 *	0.712 **	-	-	-	-	-	-
VI	−0.460	−0.616 *	0.520	0.748 **	0.892 **	-	-	-	-	-
VII	−0.772 **	−0.820 **	0.285	0.712 **	0.489	0.600 *	-	-	-	-
VIII	−0.418	−0.605 *	0.455	0.941 **	0.901 **	0.865 **	-	-	-	-
IX	0.342	0.410	−0.581 *	−0.554 *	−0.745 **	−0.637 *	-	-	-	-
X	0.581 *	0.553	−0.188	−0.266	−0.329	−0.301	0.583 *	-	-	-
XI	0.388	0.442	−0.447	−0.557 *	−0.682 *	−0.606 *	0.920 **	-	-	-
XII	0.055	0.102	−0.644 *	−0.088	−0.463	−0.464	0.638 *	0.452	-	-
XIII	0.307	0.413	−0.734 *	−0.571	−0.747 **	−0.647 *	0.886 **	0.822 **	-	-
XIV	0.088	0.162	−0.683 *	−0.380	−0.616 *	−0.622 *	0.592 *	0.483	-	-
XV	−0.054	−0.200	0.091	0.633 *	0.191	0.350	−0.135	−0.003	-	-
XVI	−0.056	−0.126	−0.065	0.552	0.167	0.425	−0.149	−0.219	0.710 **	-
XVII	0.013	−0.051	0.487	0.348	0.263	0.463	−0.248	−0.154	0.608 *	0.595 *
XVIII	0.043	−0.070	0.131	0.552	0.141	0.353	−0.121	−0.052	0.913 **	0.857 **
XIX	0.139	0.029	−0.085	0.554 *	0.143	0.338	−0.085	−0.058	0.874 **	0.922 **

**Legend:** I—Clients/patients room surface fungal load; II—Total fungal load surface; III—Total fungal load equipment; IV—Clients/patients room air fungal load; V—Optometry office air fungal load; VI—Outdoor air fungal load; VII—Total air fungal load; VIII—Total indoor air fungal load; IX—Optometry office surface bacterial load; X—Clients/patients room surface bacterial load; XI—Surface total bacterial load; XII—Bacterial load Pupilometer; XIII—Bacterial load biomicrocospe; XIV—Equipment total bacterial load; XV—Clients/patients room air bacterial load; XVI—Optometry office air bacterial load; XVII—Outdoor bacterial air load; XVIII—Total air bacterial load; XIX—Total indoor air bacterial load; * Significant correlations at a 5% significance level; ** Significant correlations at a 1% significance level.
